# Case Report: A patient with cardiac rhabdomyoma associated with embryonal developmental dysplasia of neuroepithelial tumors, presenting with recurrent cyanosis of the face and lips

**DOI:** 10.3389/fped.2025.1703713

**Published:** 2025-11-25

**Authors:** Qian Liu, Yuan Long

**Affiliations:** Western Emergency Department, Wuhan Children’s Hospital, Tongji Medical College, Huazhong University of Science &Technology, Wuhan, Hubei, China

**Keywords:** cardiac rhabdomyoma, embryonal developmental dysplasia of neuroepithelial tumors, cyanosis, cardiac ultrasound, cranial MRI

## Abstract

**Background:**

Cardiac rhabdomyoma is a benign cardiac tumor predominantly found in children and is often associated with tuberous sclerosis. Typically, this tumor is asymptomatic; however, its size and location can compromise cardiac function, potentially leading to heart failure, which may manifest as cyanosis of the lips and respiratory difficulties. Embryonal dysplastic neuroepithelial tumor is a slow-growing benign brain tumor classified as a neuroepithelial tissue tumor. It typically does not undergo malignant transformation and originates from the cerebral cortex. The concurrent occurrence of cardiac rhabdomyoma and embryonal dysplastic neuroepithelial tumor, manifesting as facial and perioral cyanosis, is relatively uncommon in pediatric patients.

**Case report:**

We present a case of a 4-year-and-4-month-old boy who was admitted for “recurrent facial and perioral cyanosis over the past year”. Prenatal four-dimensional ultrasound revealed strong echogenic foci within the ventricles, suggesting the presence of a cardiac rhabdomyoma. One year prior to admission, the child experienced recurrent episodes of facial and perioral cyanosis. Considering his medical history, the admission diagnosis was left ventricular outflow tract obstruction due to the cardiac rhabdomyoma. However, following a systematic evaluation and investigation, the definitive cause of the “facial and perioral cyanosis” was determined to be an embryonal dysplastic neuroepithelial tumor. The child subsequently underwent tumor resection, and pathological examination confirmed the preoperative diagnosis. Postoperatively, the child has remained free of facial and perioral cyanosis for over two years.

**Conclusion:**

The concurrent occurrence of cardiac rhabdomyoma and embryonal dysplastic neuroepithelial tumor is exceedingly rare. Clinicians must exercise caution when evaluating the etiology of a child's symptoms. This case emphasizes the necessity of increasing clinicians’ awareness of this rare concomitant occurrence and highlights the vital role of timely diagnosis and surgical intervention in enhancing patient outcomes.

## Introduction

Cardiac rhabdomyoma (CR) is the most common benign cardiac tumor in children (1–4), and it is closely associated with tuberous sclerosis (TS) (3). This tumor typically forms during the embryonic development period and can often be detected early via prenatal ultrasound or diagnosed before the infant reaches one year of age (3). It commonly affects the myocardium of both ventricles and the interventricular septum (3), potentially leading to fetal arrhythmias, non-immune fetal hydrops, respiratory distress, congestive heart failure, or cyanosis (2, 3). The prognosis for CR is generally favorable, as the tumor often decreases in size or may even completely resolve. Prenatal diagnosis of CR should be performed when feasible. Due to the spontaneous regression characteristic of these masses, treatment is usually symptomatic. However, surgical resection may be necessary in life-threatening situations, particularly when large tumors cause intracardiac obstruction. Dysplastic neuroepithelial tumor (DNET) is a benign neuroglial-neuronal tumor classified in the neuronal and neuronal-glial tumor group according to the 2016 revised fourth edition of the World Health Organization Classification of Central Nervous System Tumors (5). DNET is characterized by slow growth and is histologically graded as WHO grade I (6). It was first described by Daumas-Duport et al. (7) in 1988 and typically presents as “intractable epilepsy,” primarily affecting children and young adults. The onset of the disease is early, with a disease course lasting from weeks to several decades. For symptomatic cases, the most effective treatment is lesion resection. Studies have shown that complete or subtotal resection can effectively control seizures (8). Recurrences are occasionally noted, as well as differentiation into high-grade glioma either spontaneously or following radiotherapy (9). We report a case of CR concomitant with DNET. Given that both conditions can lead to facial and perioral cyanosis, particularly in the context of a known CR, careful analysis is essential to identify the true underlying cause in conjunction with the specific symptoms of the patient. This case underscores the importance of enhancing clinicians’ awareness of this rare concomitant occurrence.

## Case report

**Figure 1:**
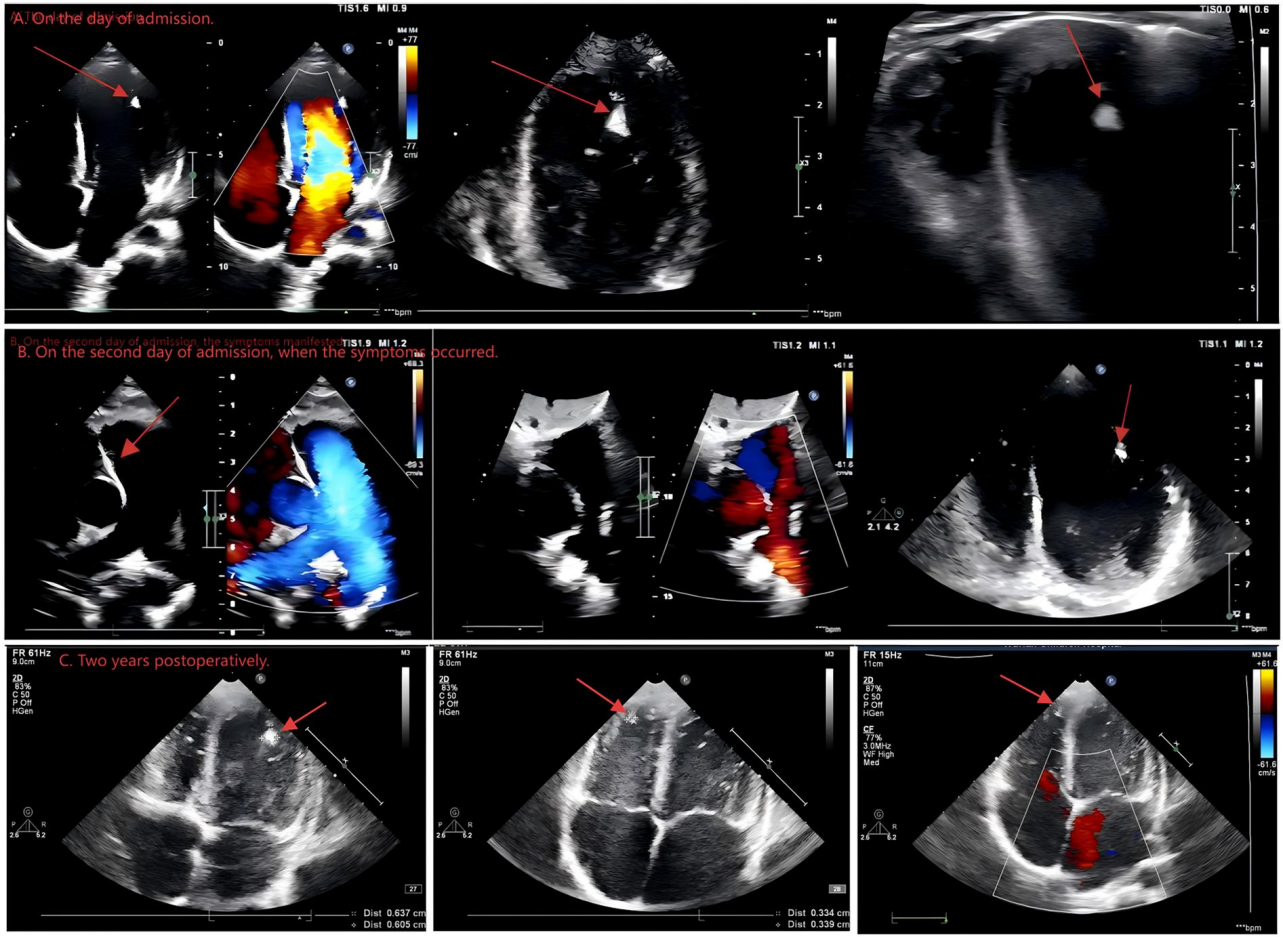
Cardiac ultrasound examination. **(A)** On the day of admission. **(B)** On the second day of admission, when the symptoms occurred. **(C)** Two years postoperatively.

A 4-year-and-4-month-old male patient presented with a chief complaint of “intermittent cyanosis around the face and lips for one year.” A fetal four-dimensional ultrasound at 28 weeks of gestation revealed a strong echogenic focus located near the right ventricular apex (largest: 12 × 9 mm), left ventricular apex (largest: 11 × 10 mm), and left ventricular outflow tract (largest: 6 × 4 mm). These masses demonstrated well-defined borders and homogeneous echogenicity, raising the suspicion of multiple CR. Subsequent fetal echocardiography also revealed multiple, well-defined, homogeneously hyperechoic masses, consistent with the imaging features of CR. Further fetal cranial magnetic Resonance Imaging (MRI) showed no definite intracranial nodules. Amniocentesis and parental peripheral blood whole-exome sequencing (WES) and copy number variation (CNV) were performed, with no pathogenic variants associated with TS complex detected. Newborn period echocardiography continued to show multiple well-defined, homogeneous hyperechoic foci in the right ventricular apex (largest: 12 × 10 mm), left ventricular apex (largest: 12 × 10 mm), and left ventricular outflow tract regions (largest: 6 × 5 mm). The patient reported no family history of tumors. In the year prior to hospitalization, the patient experienced recurrent episodes of intermittent cyanosis around the face and lips. According to parental reports, symptoms were notably exacerbated following physical activity. Each episode lasted from several seconds to 1 min, occurring 4–5 times per week. After the episodes, the patient exhibited mild fatigue but returned to baseline shortly thereafter. During the episodes, there were no significant tremors of the limbs, no fixed gaze, and the patient could occasionally respond to simple questions. Upon admission, the patient was conscious with clear mental status and brisk response. The heart rate was regular, no murmurs were auscultated over the precordium. Respiratory sounds were coarse. Limb movements were normal. Pathological reflexes were negative, and no meningeal signs were observed. The skin of the face and trunk was clear, with no rashes, plaques, pigmentation, skin lesions, or skin tags. Considering the patient's history, the outpatient physician immediately suspected that the CR might be causing cyanosis due to left ventricular outflow tract obstruction, given that the tumor could shift with cardiac motion, possibly leading to paroxysmal obstruction. Therefore, a cardiac ultrasound (Figure 1A) and electrocardiogram were promptly arranged. Echocardiography confirmed multiple hyperechoic foci in the right ventricular apex (largest: 12 × 10 mm), left ventricular apex (largest: 11 × 10 mm), and left ventricular outflow tract (largest: 5 × 5 mm). These masses demonstrated well-defined borders and homogeneous echogenicity (Figure 1A). And then the patient was admitted for continued evaluation and observation. Laboratory studies on admission, including complete blood count, liver and renal function tests, cardiac enzymes, troponin, and BNP, as well as imaging studies (chest radiography and ultrasonography of the gastrointestinal tract, liver, spleen, and both kidneys) revealed no significant abnormalities.

**Figure 2:**
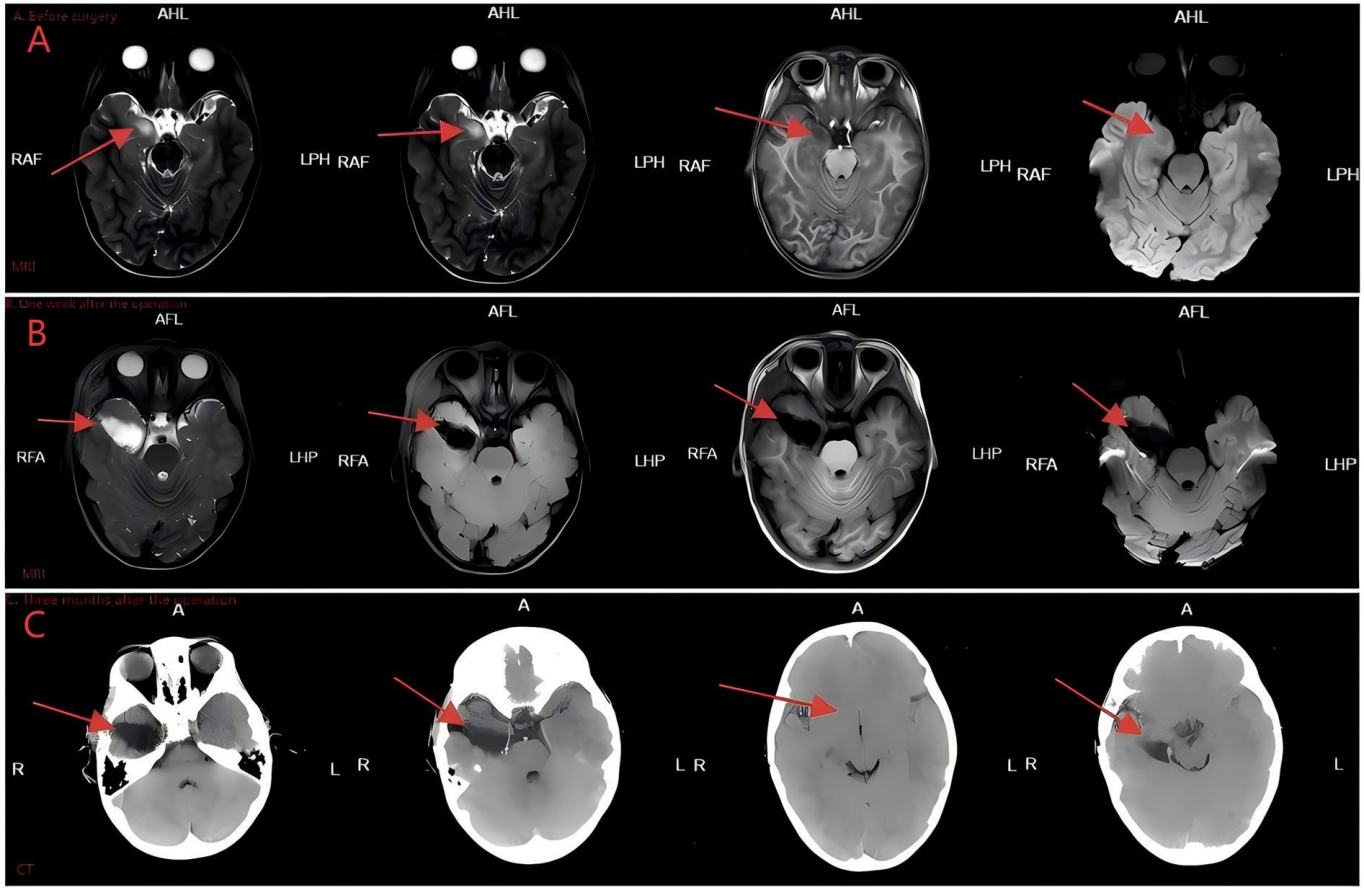
Head imaging examination. **(A)** Preoperative cranial MRI. **(B)** Cranial MRI obtained one week postoperatively. **(C)** Cranial CT scan obtained three months postoperatively.

On the second night after admission, the patient suddenly presented with symptoms including a fixed gaze, drooling at the corners of the mouth, cyanosis of the face and perioral region, slight limb tremors, and an unresponsive state, lasting approximately 3 min. During the episode, a bedside cardiac ultrasound (see Figure 1B) and bedside electrocardiographic monitoring were immediately conducted. The cardiac ultrasound during the episode did not reveal significant left ventricular obstruction, and there was no notable decrease in ejection fraction (EF). Bedside electrocardiographic monitoring showed sinus tachycardia, with no evidence of malignant arrhythmias. An experienced senior clinician promptly recognized that the paroxysmal facial and perioral cyanosis might not be attributable to the CR; consequently, the patient underwent a series of cardiovascular and neurological assessments. The patient had 24 h ambulatory blood pressure monitoring and 24 h ambulatory electrocardiogram monitoring, both of which did not indicate any significant abnormalities. A cranial MRI study, combined with arterial spin labeling (ASL) and diffusion-weighted imaging (DWI), revealed an abnormal signal in the right temporal lobe cortex, suggestive of a possible tumor lesion, such as DNET (Figure 2A). Long-term video electroencephalogram (EEG) monitoring confirmed that the focal seizures originate from the right temporal lobe region. The Griffiths Mental Development Scales (Third Edition) and the Peabody Motor Development Scales tests were both normal. Thus, the cause of the patient's paroxysmal cyanosis was ultimately identified. Following a multidisciplinary consultation with experts in cardiology, cardiothoracic surgery, and neurosurgery, the suspicion arose that the patient was experiencing seizures secondary to DNET, and surgical intervention was recommended. However, the parents had difficulty accepting the shift in diagnosis from a cardiac to a cerebral lesion and opted for pharmacological management to control the seizures. After discussions, the decision was made to initiate treatment with perampanel to manage the condition. Unfortunately, the clinical response over the following month was suboptimal.

**Figure 3:**
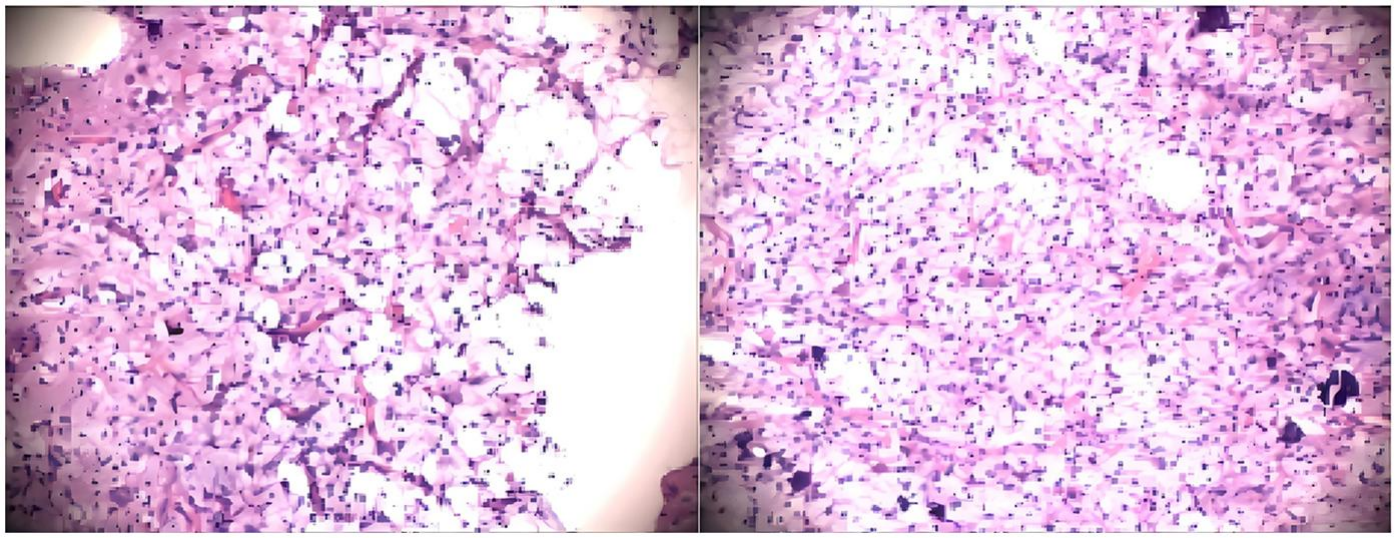
Pathological smear during the surgery.

On the 40th day after the intracranial space-occupying lesion was identified, the patient underwent resection of the temporal lobe lesion, selective amygdalohippocampectomy, and epidural drainage. Intraoperatively, a tumor was visualized at the junction of the inferior temporal gyrus and the occipitotemporal sulcus. It was relatively soft to slightly firm in texture and pale red in color. The results of the intraoperative pathological examination are detailed in Figure 3. A frozen section analysis confirmed the diagnosis of a (right temporal lobe) DNET (WHO Grade I). Immunohistochemical findings indicated: S-100 (+), GFAP (+), Syn (+), NSE (+), CD34 (−), NeuN (+), SSTR2 (+), ENA (−), Oligo-2 (+), Nestin (+), MBP (−), NF (+), Ki-67 (5% index), IDH (−), and P53 (scattered +). The intraoperative pathology confirmed the preoperative diagnosis. The patient was discharged without complications on the 11th postoperative day and was followed up in our outpatient clinic for 2 years, during which no recurrence of facial and perioral cyanosis was noted. After the operation, the patient underwent cranial imaging studies, including MRI (Figure 2B) and CT (Figure 2C), both of which indicated a favorable prognosis. Furthermore, the patient continued follow-up in the cardiology and cardiothoracic surgery outpatient clinics. The latest echocardiogram still indicated multiple hyperechoic foci in the right ventricular apex (largest: 12 × 10 mm), left ventricular apex (largest: 10 × 10 mm), and left ventricular outflow tract (largest: 5 × 5 mm), showing no significant change from the findings on admission (Figure 1C). Throughout the two-year follow-up, the child underwent three prolonged EEG monitoring sessions, none of which revealed abnormal epileptiform activity. The child has now entered primary school, with height, weight, intellectual development, gross motor function, and fine motor function all comparable to those of same-age peers.

## Discussion

CR is often associated with brain TS, sebaceous adenomas, and hamartomas of the kidneys and other organs. It commonly serves as an initial symptom of TS, prompting the diagnosis of TS before any changes are noted on skin or brain scans; this lesion is linked to mutations in the TSC1 and TSC2 genes (2). When feasible, prenatal diagnosis of CR should be performed, particularly in any family with a history of TS, and thorough genetic counseling is essential (10). Similarly, any fetus found to have a cardiac tumor on ultrasound requires evaluation of other family members for TS (10). In this case, prenatal screening suggested the possibility of CR, prompting a comprehensive prenatal evaluation for TS complex. This evaluation included fetal echocardiography, fetal cranial MRI, amniocentesis, and parental blood sampling for WES and CNV analysis. Testing revealed that neither the fetus nor the parents carried known TS complex-related pathogenic variants, and no clinical signs of TS complex were observed in the parents. Given the known potential for spontaneous regression of isolated CR, the parents decided to continue the pregnancy after thorough genetic counseling. Cardiac ultrasound during both the neonatal period and after admission revealed multiple, solid, hyperechoic masses in the left ventricular wall and outflow tract, without calcification or pericardial effusion—findings consistent with CR. This series of results initially created a diagnostic dilemma, leading to the erroneous conclusion that the paroxysmal face and lips cyanosis was due to a cardiac mass effect. During hospitalization, the child underwent a systematic evaluation. Repeated echocardiograms ruled out other cardiac tumors, such as typically solitary and calcified ventricular fibromas, or pericardial teratomas, which often present with pericardial effusion and tamponade. A full systemic assessment—including evaluation of the skin, kidneys, and neurodevelopmental status—also showed no abnormalities, further supporting the absence of TSC-related manifestations. The diagnostic impasse was ultimately broken by a seemingly incidental generalized tonic-clonic seizure, which provided the crucial clue leading to identification of the true underlying etiology.

DNET is a benign glioneuronal tumor primarily affecting children and young adults with chronic epilepsy. Its main characteristic is the presence of a granular layer located within the cortex (11). DNET lesions are often associated with focal cortical dysplasia (FCD) type IIIb, which is considered to play a significant role in the pathogenesis of epilepsy. The pathophysiological and molecular mechanisms underlying DNET development remain unclear, but it is generally believed to have favorable surgical treatment outcomes and prognosis (7, 12). However, a limited number of reports exist regarding cases of DNET that initially appeared benign but later transformed into malignant tumors (9, 13). The malignant transformation seems to be relatively exceptional, with initially benign lesions potentially exhibiting more aggressive characteristics (14). The time interval for recurrence after initial resection can vary from several months to several years (14). DNET resection can effectively stabilize the clinical condition of patients. A meta-analysis indicated that the prognosis of seizures in patients with DNET or ganglioglioma is influenced by the duration, type, and extent of seizures (15). Factors associated with poor seizure prognosis include secondary generalized seizures, epilepsy duration exceeding one year, and subtotal resection of the lesion (15). Another data analysis showed a significant statistical correlation between shorter preoperative epilepsy duration and better prognosis (16). In this case, the patient underwent surgery on the 40th day following the diagnosis of the underlying condition. Postoperative follow-up with cranial MRI and CT revealed that the patient had a favorable recovery. During the outpatient follow-up period exceeding two years, the patient did not experience any recurrence of cyanosis around the face and lips or transient loss of consciousness, demonstrating a favorable prognosis, which is consistent with previously published reports. The favorable outcome postoperatively is closely related to factors such as timely identification of the underlying cause, the short duration of seizure symptoms, the absence of microglial proliferation on immunohistochemical profiling, and the complete resection of the lesion.

CR and DNET are generally regarded as distinct clinical entities. While CR demonstrates a well-established association with TS (3), the latter disorder can lead to a spectrum of cerebral lesions ranging from focal cortical dysplasia to potential neoplastic pathologies such as subependymal giant cell astrocytoma (SEGA) (17, 18). These neurological manifestations are typically characterized by drug-resistant epilepsy and intellectual disability (19). The uniqueness of the present case lies in the co-occurrence of an isolated CR—without detected TS-related pathogenic variants or typical clinical features—and a cerebral DNET. The child's primary manifestation was face and lips cyanosis, while neurological development—including gross motor function and intellectual capacity—remained within normal limits, constituting a clinically rare scenario. To our knowledge, no previous literature has systematically discussed the co-occurrence of CR and DNET. We report this rare case to provide insights for the diagnosis and management of similarly complex clinical presentations in the future.

## Conclusion

CR and DNET are both common benign tumors in children; however, their concurrent occurrence is extremely rare. Since both conditions can lead to cyanosis around the face and lips, particularly in cases where cardiac rhabdomyoma has been diagnosed, clinicians should exercise caution when evaluating the underlying causes of the child's symptoms. A thorough analysis of the specific symptoms is essential to identify the true etiology. This case underscores the importance of increasing clinical awareness regarding this rare concomitant presentation, while also highlighting the critical role of timely identification of the underlying cause and surgical intervention in improving the prognosis for the patient.

## Data Availability

The original contributions presented in the study are included in the article/Supplementary Material, further inquiries can be directed to the corresponding author.
